# Low frequency mechanical actuation accelerates reperfusion in-vitro

**DOI:** 10.1186/1475-925X-12-121

**Published:** 2013-11-21

**Authors:** Marcin Marzencki, Behrad Kajbafzadeh, Farzad Khosrow-khavar, Kouhyar Tavakolian, Maxim Soleimani-Nouri, Jaap Hamburger, Bozena Kaminska, Carlo Menon

**Affiliations:** 1 1Faculty of Appliced Science, Simon Fraser University, 8888 University Drive, Burnaby, BC, Canada; 2 Vancouver General Hospital, 855 12th Avenue West, Vancouver, BC, Canada

## Abstract

**Background:**

Rapid restoration of vessel patency after acute myocardial infarction is key to reducing myocardial muscle death and increases survival rates. Standard therapies include thrombolysis and direct PTCA. Alternative or adjunctive emergency therapies that could be initiated by minimally trained personnel in the field are of potential clinical benefit. This paper evaluates a method of accelerating reperfusion through application of low frequency mechanical stimulus to the blood carrying vessels.

**Materials and method:**

We consider a stenosed, heparinized flow system with aortic-like pressure variations subject to direct vessel vibration at the occlusion site or vessel deformation proximal and distal to the occlusion site, versus a reference system lacking any form of mechanical stimulus on the vessels.

**Results:**

The experimental results show limited effectiveness of the direct mechanical vibration method and a drastic increase in the patency rate when vessel deformation is induced. For vessel deformation at occlusion site 95% of clots perfused within 11 minutes of application of mechanical stimulus, for vessel deformation 60 centimeters from the occlusion site 95% percent of clots perfused within 16 minutes of stimulus application, while only 2.3% of clots perfused within 20 minutes in the reference system.

**Conclusion:**

The presented in-vitro results suggest that low frequency mechanical actuation applied during the pre-hospitalization phase in patients with acute myocardial infarction have potential of being a simple and efficient adjunct therapy.

## Introduction

Myocardial infarction and stroke (arterial thrombosis) comprise the leading causes of death and disability in the developed world [1]. Emergency percutaneous coronary intervention is the preferred treatment method for acute myocardial infarction, but emergency iv thrombolysis still plays an important role [2,3]. This is especially true in remote areas, where the geographic distance to hospitals with a cardiac catheterization laboratory eliminates the possibility of fast treatment. As the delay in blood flow restoration at the occlusion site is the main factor of clinical outcome [4], rapid treatment after the onset of symptoms is crucial. Late presentation to an angioplasty facility and frequent incomplete reperfusion following iv thrombolysis have stimulated research for alternative or adjunctive emergency therapies that could be initiated prior to patient arrival to hospital. Ultrasound assisted thrombolysis using catheter mounted transducers has been investigated and there is evidence that it accelerates the rate of thrombolysis [5,6] and increases the penetration of lytic enzymes into the thrombus [7,8]. Based on these results, non-invasive transcutaneous application of ultrasound was investigated. Initial clinical evaluation in patients with acute myocardial infarction confirm the safety of application when pulsed ultrasound was used to avoid excessive tissue heating [9]. Nevertheless, subsequent clinical trials of thrombolysis assisted by externally applied ultrasound failed to prove significant improvement in reperfusion rates [10]. Furthermore, accurate placement of the ultrasound transducer was deemed crucial to treatment efficacy eliminating the possibility of application by untrained personnel [10]. Finally, it has been discussed that lower frequency ultrasound, offering better tissue penetration, provides better results [6,8]. An intragastral resonator delivering low frequency mechanical waves has been reported to disrupt big blood clots, tablets, food, or scybala and was proposed as a possibility during endoscopic examinations [11]. Furthermore, Folts [12] reported clearing of thrombosed arteries in an open animal model when external tapping was applied directly to the occlusion site. Lower frequency mechanical vibration offers better tissue penetration and can be applied to the external chest wall of the patient, and therefore has been proposed as a safe and effective substitute to ultrasound [13-16]. We hypothesize that such a mechanical approach could be a safe and effective adjunct method to clot disruptive drug therapy, thereby accelerating reperfusion of thrombolytically occluded vessels. External mechanical actuation can be started in the field by minimally trained emergency personnel or even by bystanders immediately after the onset of symptoms. It has been hypothesized that application of mechanical stimulus can increase mixing of thrombolytic drugs in patients bloodstream, thus improving drug penetration to the occlusion site. Therefore, lower doses of thrombolytic drugs could be used during pharmacological therapy, which might result in a lower risk of bleeding. This could be of clinical relevance, specifically for patients with a relative contraindication for thrombolytic drug use [13,14].

Yohannes and Hoffmann [14] studied the impact of mechanical vibrations applied through an attenuating barrier on re-canalization of a thrombosed flow system. They concluded that localized transcutaneous low frequency mechanical vibration might serve as a safe and practical method to accelerate clot dissolution. Their study had some limitations, including: 

• The model for stenosis site was vessel deformation, rather than a narrowing, so the actual lumen area remained unchanged.

• The pressure was gravitationally applied instead of creating a pressurized flow system and the actual frequency and amplitude of the mechanical actuation induced was assumed, not measured.

• The vibratory stimulus was applied directly over the occlusion, the effect of improper device placement was not investigated.

Previous studies have established the beneficial effect of mechanical actuation on accelerating reperfusion. However, the exact mechanism of actuation of the thrombosed vessel resulting from transcutaneous application of mechanical vibrations has not been explored in detail. Externally applied vibration can both induce vibration of the blood carrying vessels and their deformation. Even though it has been stated that low frequency vibration penetrates tissue better than ultrasound alleviating the importance of device placement precisely over the occlusion, no research to date has explored the efficacy of stimulus application remotely from the clot.

The goal of this study is to verify the effectiveness of various methods of low frequency mechanical actuation in accelerating in-vitro reperfusion. To this end, we studied a stenosed, partly occluded heparinized flow system subject to arterial-like pressure variations with one of three actuation methods applied: direct vibration of the occlusion site, deformation of vessel 20 mm proximal to the occlusion sites and deformation of a larger vessel 60 cm distal to the occlusion sites. We hypothesize that vibration of vessels promotes mixing and penetration of anti-thrombolytic agents in the bloodstream which could lead to accelerated reperfusion. Vessel deformation on the other hand, induces local pressure variations that result in both increased mixing of drugs in blood and mechanical deformation of the blood clot and thus its accelerated disintegration. These hypotheses are herein investigated.

## Materials and method

The experiments described in this paper were performed at the University of British Columbia Farm, 6182 South Campus Road, Vancouver, BC, Canada, in accordance with ethics approval 2009s0630.

### Flow system

**Figure 1:**
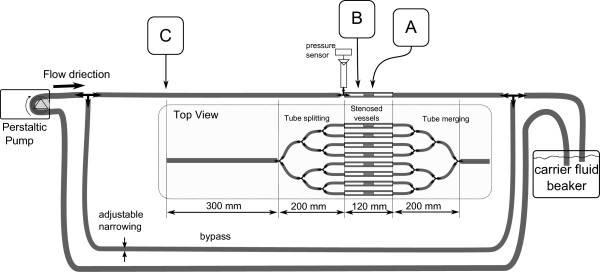
**The flow system.** Schematic representation of the experimental setup used in this study with **A**, **B**, and **C** indicating the location of the corresponding actuation sites.

**Figure 2:**
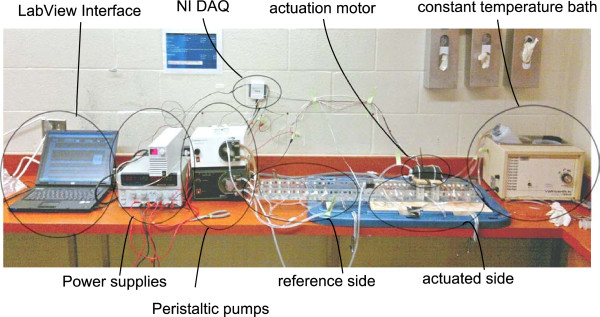
Experimental setup, photograph of the experimental setup used in this work.

A schematic of the setup prepared for this experiment is shown in Figure 1 and a photograph is presented in Figure 2. It is intended to model a stenosed arterial system subject to systemic pressures. It consists of a heparinized flow system and one of three actuation setups. A rolling peristaltic pump (Manostat 72-300-000) induces flow of a buffer fluid (heparinized saline solution) through a tubing system and through eight narrowings modeling the stenosis sites. The fluid was aggregated in a constant temperature bath (Vanlab, VWR Scientific, Inc) and kept at 37°C. For all experiments, a reference setup identical to the test setup but absent the actuation elements was used to provide a baseline result. The two setups (i.e. the reference and the actuated one) shared the buffer fluid in the beaker. Tubing types used to build the flow system were chosen to be similar in size and mechanical properties to human arteries [17]. Types, dimensions, and properties of the tubing chosen for each element of the system are reported in Table 1. A bypass was installed to provide a path around the occlusion sites to simulate more closely an actual obstruction in a living organism, where not all vessels are blocked simultaneously. The lumen of the bypass was adjustable to control the flow rate and the induced pressure. The total capacity of the tubing was between 165 ml and 200 ml depending on the actuation system used. The fluid pressure was measured at the edge of one of the narrowings, as shown in Figure 1, using a pressure sensor (MPXM2053GS, Freescale Semiconductor, Inc, Austin, TX) connected to an instrumentation amplifier (INA110, Texas Instruments, Dallas, TX). A bi-axial MEMS accelerometer (ADXL278, Analog Devices, Inc., Norwood, MA) was used to measure motion induced at the actuation site.

**Table 1:** Materials used to construct the flow system and the clot formation setup

**Vessel**	**Material**	**Length**	**ID [mm]**	**OD [mm]**	**Durometer**	**P/N**	**Brand**
Connectors	Silicone	2m	4.76	7.95	50A	SMD-188A-25	X-Med
Clot formation	Silicone	30 cm	4.76	7.95	50A	SMD-188A-25	X-Med
Narrowing outside	PVC	120 mm	4.76	6.35	66A	TMT-187A-50	Tygon
Narrowing inside	PVC	15 mm	1.59	4.76	66A	TMT-062B-10	Tygon
Large tube	PVC	120 mm	9.5	12.7	55A	8000-0120	T. Scientific

Data acquisition was done in the LabView environment using a custom made Virtual Instrument and a data acquisition module (NI USB-6009 DAQ, National Instruments, Austin, TX). Continuous data acquisition was performed throughout the experiment at a sampling rate of 1 kHz and the acquired data was stored in a text file on a personal computer.

During the experiment, flow in the system was regulated using the adjustable narrowing on the bypass to 2 ml/s and pressure varying between 25 kPa and 30 kPa (188 mmHg and 225 mmHg respectively).

#### Stenosis sites

**Figure 3:**
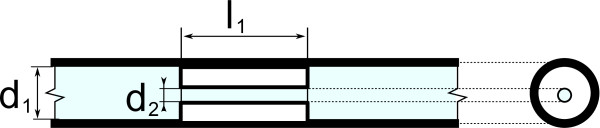
**Construction of the narrowing.** Stenosis site construction, where *d*_1_=4.76 *mm*, *d*_2_=1.59 *mm* and *l*_1_=15 *mm*.

There was a total of 16 narrowings in the experimental setup used to model stenosis sites, eight for the reference side and eight for the actuated side. Each narrowing in a given setup was identical and created by inserting a 15 mm plastic tube element (narrowing inside in Table 1) into a larger plastic tube (narrowing outside in Table 1), as depicted in Figure 3. In order to insert the smaller tube, the larger tube was cut in half and then glued around the smaller tube with cyanoacrylate glue (Loctite Pro Liquid) and sealed with a silicon glue-sealant (Dow Corning 732). Finally, the tube was cut into proper length and desired proportions before and after the narrowing with a total length of 120 mm. The resulting structure presented a 90% luminal stenosis. Installation of narrowings in the actuated and the reference systems was randomized. The internal diameter and length of the tubing was chosen to correspond to the internal diameter of the human coronary artery, based on the middle section of the right coronary artery [18].

#### Buffer fluid

The buffer fluid used in the system was a solution of 2000 Units of heparin (H3393, Sigma-Aldrich, Saint Louis, MI) in 1000 ml of 0.9% Sodium Chloride IV injection USP solution (Baxter International, Deerfield, IL). The fluid was brought to 37°C prior to the experiment and kept at a constant temperature. We used very low concentrations of 2 USP units of heparin per ml, much below the amount of heparin needed to prevent coagulation in whole blood which is 20 to 50 units per mL of whole blood [19].

#### Actuation

**Figure 4:**
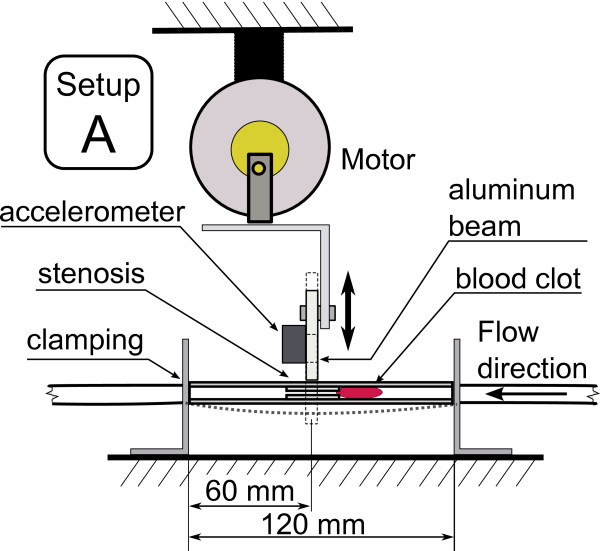
**In-vitro setup A.** Actuation setup A where the stenosis sites are directly vibrated.

**Figure 5:**
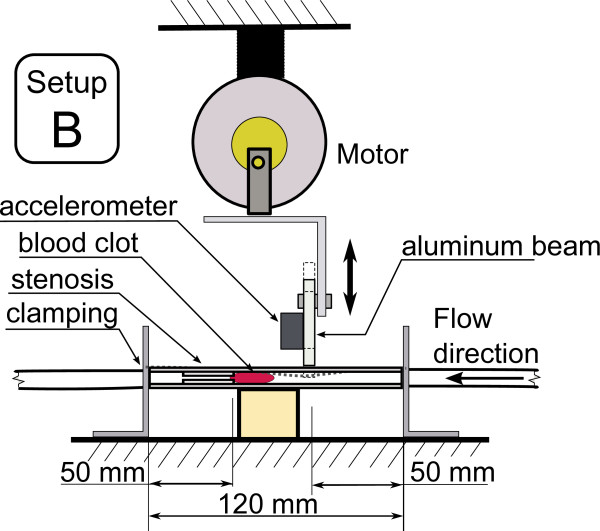
**In-vitro setup B.** Actuation setup B where mechanical deformation is applied to the vessel proximal to the stenosis site.

**Figure 6:**
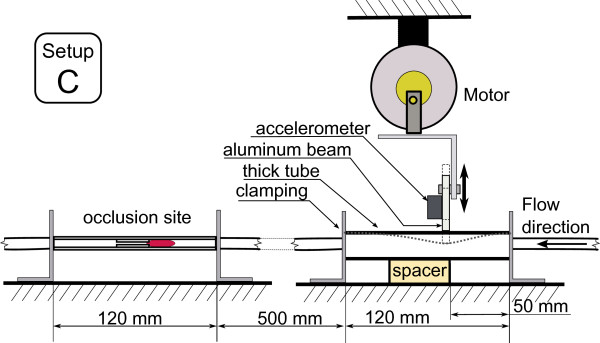
**In-vitro setup C.** Actuation setup C where mechanical deformation is applied 60 cm from the stenosis site to a larger vessel.

**Figure 7:**
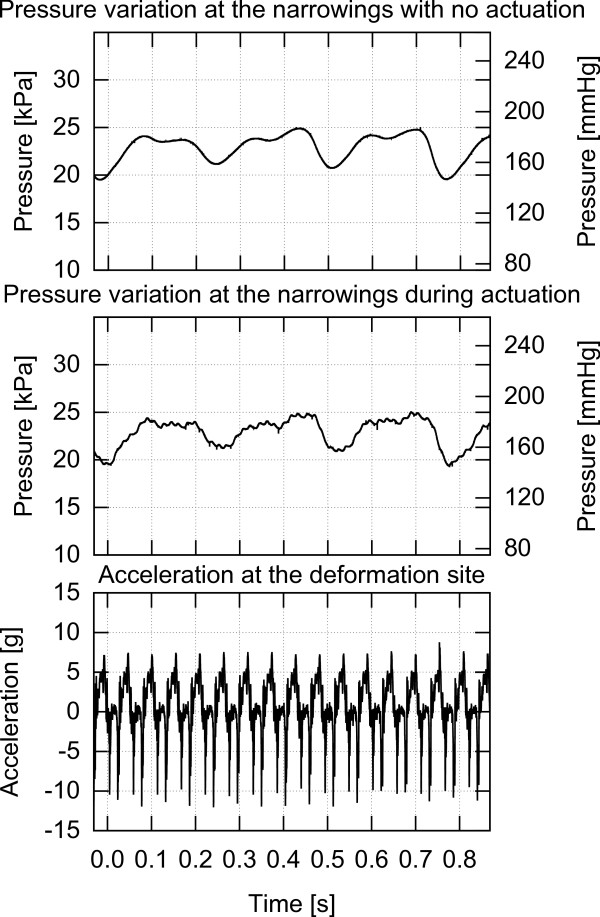
**Pressure and acceleration, setup A.** Pressure in the flow system with and without mechanical stimulus and acceleration measured at the actuation site while using setup A.

**Figure 8:**
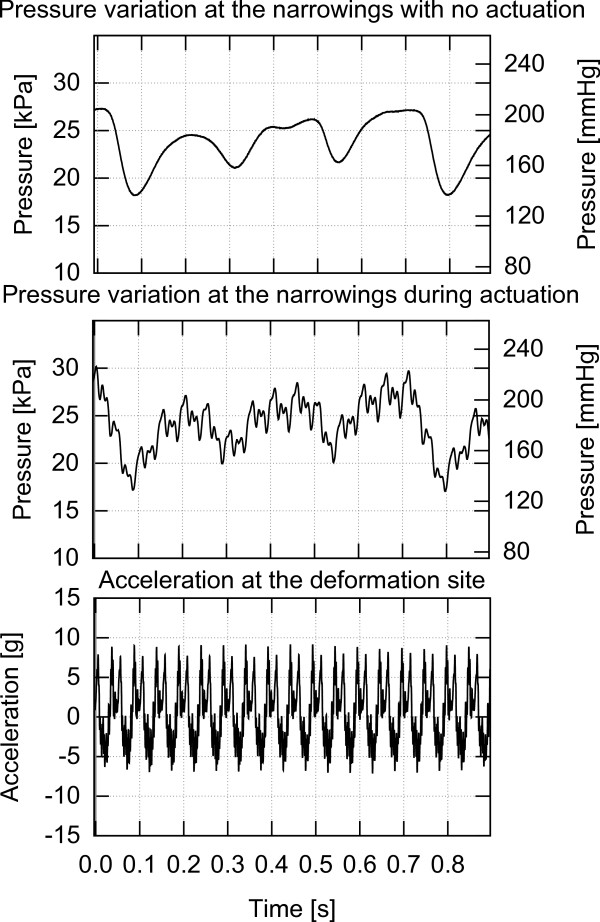
**Pressure and acceleration, setup B.** Pressure in the flow system with and without mechanical stimulus and acceleration measured at the actuation site while using setup B.

**Figure 9:**
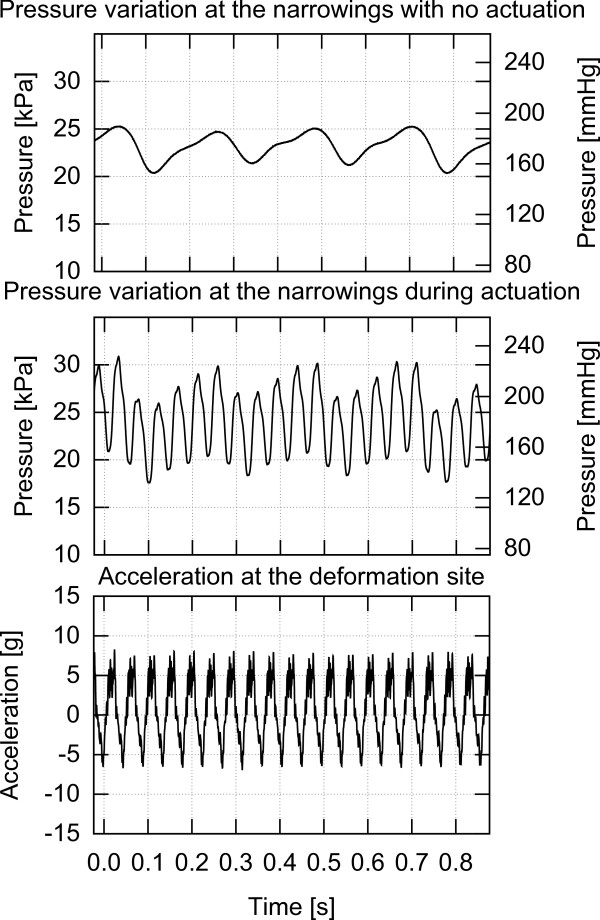
**Pressure and acceleration, setup C.** Pressure in the flow system with and without mechanical stimulus and acceleration measured at the actuation site while using setup C.

This work investigates the effect of various types of low frequency mechanical actuation on accelerating vessel reperfusion. In each experiment, one of the three actuation setups described below and depicted in Figures 4, 5 and 6 was used and positioned as depicted in Figure 1. The resulting pressure at the narrowings with no actuation, pressure during actuation and the acceleration at the application site are shown in Figures 7, 8 and 9 for each of the three corresponding setups from Figures 4, 5 and 6. A: The stenosis sites were directly vibrated at 5 g amplitude (2 mm displacement at 24 Hz). The mechanical stimulus was applied at the occlusion site and the lumen of the vessel was not directly deformed. B: Deformation was applied to the occluded vessel 20 mm before each stenosis site. In this case, the vessel was directly deformed and the clot was placed between the narrowing and the application site, but was not directly deformed. The vessel was reaching 80% focal occlusion at the deformation site during actuation. C: Deformation was applied to a vessel with an inside diameter larger than the one of the stenosed vessels, corresponding to the diameter of human aorta in the abdominal region [17]. The actuation site was placed 60 centimeters from the stenosis site. In this case, no mechanical stimuli was applied directly to the narrowings. The amplitude of deformation applied to the large vessel was equal to 3 mm.

In all the three cases, the mechanical movement was generated by a DC motor (Unionwell LW2920D120) with shaft extending from both ends coupled with an aluminum beam through a lever system implying a quasi linear motion. The motor location could be adjusted horizontally and vertically to accommodate various configurations of distances. The aluminum beam was either coupled with a free moving narrowing (setup A) or induced a direct deformation of the vessel immobilized by a wooden spacer (setups B and C). In cases A and B, the actuation was close to the blood clot, and in the case C remote from the clot. The induced displacement was constant independent of frequency. The actuation frequency was controlled through the DC voltage supplied to the motor and observed using an accelerometer (AD22286, Analog Devices, Cambridge, MA) mounted on the aluminum beam. For all the three experiments, the actuation frequency was between 20 Hz and 24 Hz.

### Experimental procedure

A total of 198 occlusions was successfully tested in all experiments with 27 occlusions subject to direct vibrations, 56 subject to local vessel deformation, 37 subject to remote vessel deformation and the remaining 78 providing the baseline result. A total of 28 experiments was discarded due to occlusion instability (immediate perfusions).

#### Clot preparation

**Figure 10:**
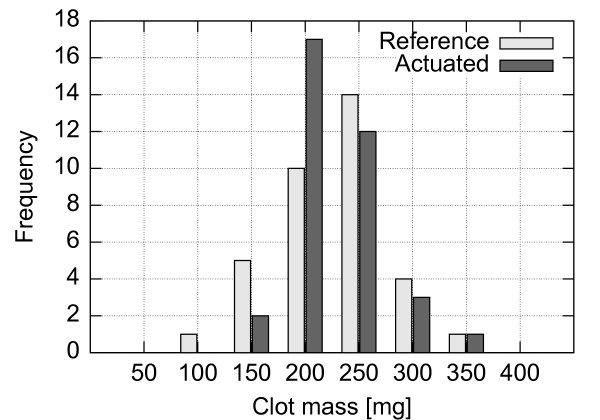
**Histogram of initial clot masses for setup A.** Distribution of clot masses prior to the experiments using the setup A.

**Figure 11:**
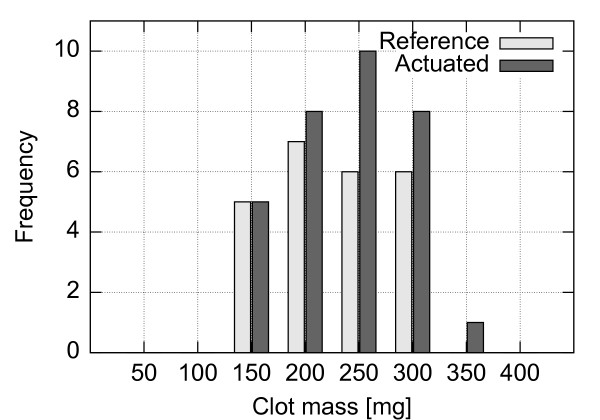
**Histogram of initial clot masses for setup B.** Distribution of clot masses prior to the experiments using the setup B.

**Figure 12:**
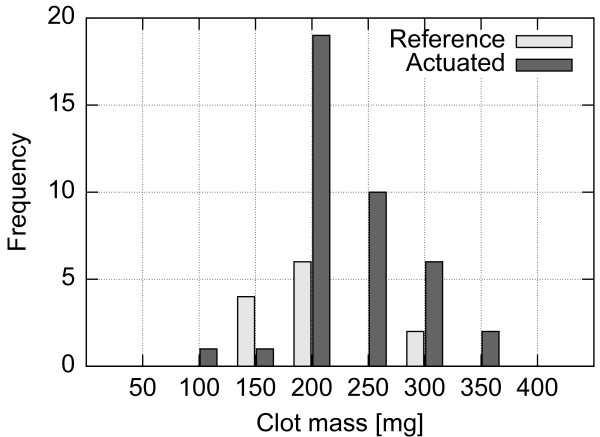
**Histogram of initial clot masses for setup C.** Distribution of clot masses prior to the experiments using the setup C.

Whole blood was collected from the left jugular vein of a sheep (female, Ovis aries, Suffolk breed) elevated in a controlled environment at UBC Farm. Blood was drawn under standardized conditions into a 60 ml syringe and immediately distributed into sterile silicon tubing (ID 4.76 mm), sealed inside a double zip-lock bag and placed into a water bath held at 37°C. Ninety minutes from blood extraction, the clotted blood was removed from the bath and inspected visually for retraction. The retracted clot was pressed out of the silicon tube and verified for uniformity. The long columnar clot was then cut into 15 mm pieces and each piece was weighted separately using a digital scale (VB-302A, Virtual Measurements & Control, Santa Rosa, CA). The resulting distribution of clot weights in each experiment is detailed in Figures 10, 11 and 12. Cut and weighted clots were immediately inserted into the flow system by disconnecting the narrowings separately one by one at clamping, placing a clot inside the tube with a spatula and subsequently flooding it with the fluid from the beaker and reconnecting the narrowing. Upon re-pressurization the clots occluded the closest stenosis site. Special care was taken to remove all air bubbles from the system prior to pressurizing and not to damage the clots mechanically. The clot distribution between the stenosed channels and the reference versus the actuated setups was randomized.

#### Flow system

In order to verify occlusion stability, after insertion of all clots, the system was pressurized using the peristaltic pump for 120 seconds without applying actuation. Narrowings where clots perfused within this period were clamped and labeled as immediate perfusions. The reference and the actuated setups were functionally identical during this period. Throughout all the experiments, 12.23% of clots perfused prior to actuation (immediate perfusions).

#### Actuation

After the initial test for 120 seconds, the actuation setup was activated. Perfusions were detected through abrupt reduction of the buffer fluid pressure at the narrowings. Each perfused narrowing was immediately clamped to maintain constant pressure in the system. The time to perfusion was noted from the activation of actuation. After 20 minutes from the start of actuation, the system was stopped and all the remaining clots (that did not perfuse) were removed, drained and re-weighted.

## Results

The three setups described in the previous section were evaluated for their efficiency in accelerating reperfusion of occluded vessels.

### Setup A

In this case, the stenosis sites were directly vibrated. The pressure variations at the occlusion sites both prior to actuation and during actuation along with acceleration during actuation are presented in Figure 7. It can be seen that the mechanical vibrations have minimal impact on the fluid pressure at the narrowings.

**Figure 13:**
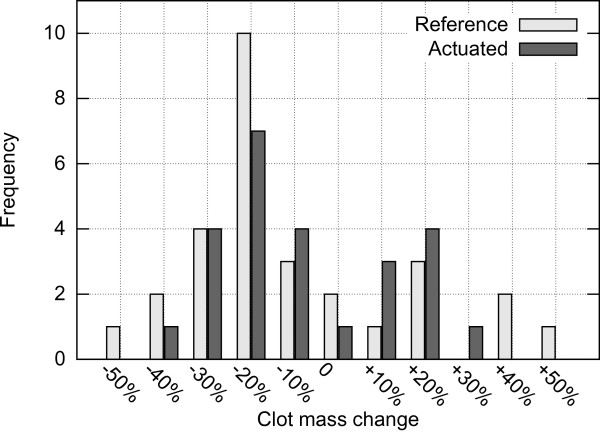
**Clot mass change.** Clot mass change post experiment versus prior to experiment when using setup A. Negative percentage value signifies reduction in clot mass.

Out of 35 tests, only 1 perfusion occurred within the duration of actuation (20 minutes) after 13 minutes on the actuated side resulting in a TIMI 2 flow. Therefore, only the change in the clot masses was evaluated. Figure 13 presents a histogram of clot mass changes evaluated as the difference between the clot mass post experiment versus the clot mass prior to the experiment. The average mass change is -15.89% (*σ*=26.4%) for the reference system and -12.16% (*σ*=19.1%) for the actuated system. The fact that mass of some clots increased may be due to water absorption, or imperfect soaking method employed.

### Setup B

In this case, a direct mechanical deformation of the vessel was imposed near the stenosis site. Figure 8 presents the pressure variations prior to actuation and during actuation along with acceleration at the application site while using setup B to impose vessel deformation. It can be seen that vessel deformation is inducing significant pressure variations at the occlusion site and thus at the blood clot. Contrary to the case where direct vibrations were applied, in the deformation case over 95% of the narrowings were successfully perfused resulting in a TIMI 3 flow within 11 minutes of the onset of actuation while only two perfusions (5.3%) occurred on the reference side within 20 minutes.

### Setup C

In this case, a direct mechanical deformation was imposed 60 centimeters from the stenosis sites to the fluid carrying vessel with a larger ID.

Figure 9 presents the pressure variations at the narrowings with and without actuation and the acceleration at the application site while using setup C. It can be seen that as in the case of using setup B, there are significant pressure variations at the occlusion site induced by the actuation. As a result of actuation induced using setup C, over 95% of narrowings perfused within 16 minutes of application resulting in a TIMI 3 flow, whereas there was no perfusion on the reference side. Only one occlusion out of 37 actuated narrowings did not perfuse within 20 minutes.

**Figure 14:**
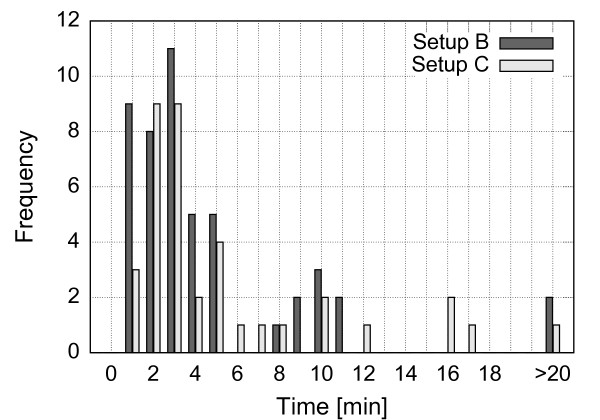
**Times to perfusion.** Histogram of times to perfusion while using setup B or setup C to induce vessel deformation.

**Figure 15:**
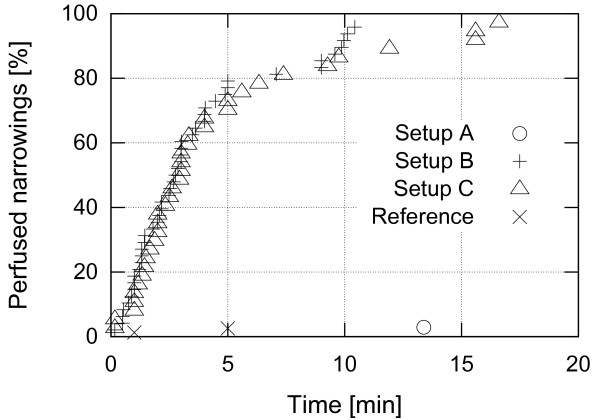
**Perfusion rate.** Reperfusion rate among the tested setups for the first 20 minutes of actuation.

Setups B and C have proven to be the most efficient in accelerating reperfusion. Figure 14 presents distribution of times to perfusion for the actuated side for those setups. The average time to perfusion was 3 minutes 50 seconds for setup B and 4 minutes 27 seconds for setup C. Figure 15 summarizes the patency rate for all the experiments. It can be seen that setups B and C have very similar distribution of times to perfusion which indicates that deformation of a large vessel distant from the occlusion site is equally efficient as deformation of vessels very close to the occlusion site. On the other hand, patency rate for setup A is very close to the baseline.

## Discussion

Our study evaluates the effectiveness of various actuation methods in accelerating re-perfusion of occluded vessels in an in-vitro model. The experimental results show that while keeping pressure in the system at systemic levels, 95% of occlusions perfuse within 11 minutes of application in the case of a mechanical deformation applied at the occlusion site and 95% of the occlusions perfuse within 16 minutes of application if a mechanical deformation is applied 60 centimeters from the occlusion site. Contrary to indications from previous publications, we demonstrated that direct vibration of the occlusion sites has no significant impact on accelerating reperfusion.

**Figure 16:**
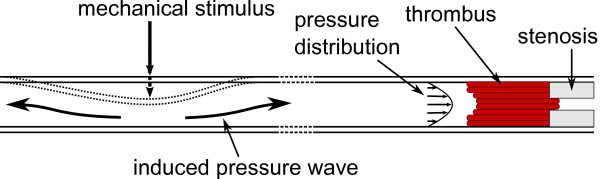
**Pressure distribution.** Schematic representation of pressure distribution in a vessel induced by vessel deformation distant from stenosis site.

The notion that mechanical actuation positively affects the speed at which a blood thrombus disintegrates was suggested in previous publications. However, our study is the first to systematically evaluate the various proposed application methods. The three approaches examined in this study apply either displacement or deformation at specific frequencies. We theorized that vessel vibration would induce more mixing in the buffer fluid and promote clot lysis, but our experimental results prove otherwise. No significant improvement in reperfusion time was observed when occluded vessel was directly vibrated. It may be due to the very narrow lumen of the vessels and the lack of pressure waves that would deform the clot and stimulate mixing. On the other hand, we demonstrated that pressure variations induced by vessel deformation drastically improve reperfusion rate. Mechanical stimuli create pressure waves that are transferred by the fluid to the occlusion site. If deformation is applied distant to the occlusion site, the amplitude of pressure variations is reduced due to compliance of vessels (albeit low for aortic walls). Still, as the blood is a non-compressible fluid, the pressure waves are usually well transferred through the circulatory system of a living organism [20]. Fluid viscosity and its traction on the vessel walls imply pressure non-uniformity in the lumen of the vessel, as depicted schematically in Figure 16, which can have further positive impact on clot dissolution. If the central part of the thrombus is subject to higher forces than the outer parts, then clot deformation is more prominent and lysis is further promoted. Furthermore, arterial thrombus is often characterized by presence of alternating layers called Lines of Zahn [21-23]. Lines of Zahn are characteristic of thrombus formed at rapid arterial blood flow, like the one in the coronary arteries, where laminations are produced by deposition of platelets and fibrin (pale layers) and red blood cells (dark layers). A blood clot presenting such a layered construction would be much more prone to deformations induced by the nonuniform pressure distribution applied at the front of the thrombus.

During our experiments, we observed that after perfusion the blood clot is split into multiple long and narrow fragments that easily pass through occlusion sites when recirculated in the system. It can be argued that in-vivo such fragments may occlude further arteries leading to a “no reflow” situation known from unsuccessful angioplasty procedures. Nevertheless, in the presented method, the fragments of disintegrated clot are sufficiently large not to spread across all capillaries in the myocardial muscle. Therefore, any displacement of the occlusion site down the bloodstream would enhance delivery of the oxygenated blood thus reducing the extent of myocardial muscle death and increasing the chances of patient survival.

The fact that even distant vessel deformation accelerates clot lysis opens way to application of mechanical stimuli away from the chest area of patients suffering from acute myocardial infarct. Furthermore, as it is not critical to apply the stimulus directly over the occlusion site, contrary to ultrasound, lack of exact knowledge of the occlusion site location does not exclude efficient treatment, as long as a major artery is targeted. A feedback system, where a high frequency blood pressure monitor would detect pressure waves in an easily accessible artery (e.g. thyroid), could be used to verify the efficiency of application. Thus, such method could be effectively applied in the field using a simple external massager device operated by minimally trained personnel. Finally, as the chest area is very sensitive, any prolonged mechanical deformation is undesirable, contrary to the more robust abdominal section containing large blood carrying vessels, like the aorta. In order to induce a displacement of 3 mm in amplitude at the aorta level, a significant displacement has to be applied at the surface of the body. Our tests indicate that peak to peak displacement of 8 mm at 30 Hz applied using a commercial massager (Max F-209, Brookstone, Merrimack, NH) can be tolerated in the abdominal area for at least 20 minutes with an application force of 50 N. The in-vitro results presented in this paper indicate that such application would be sufficient to promote reperfusion in case of acute myocardial infarction.

The limitations of our study mostly consisted in the level of fidelity with which the in-vitro system represents an actual circulatory system. We studied the effect of various methods of application without analyzing the mechanism of mechanical stimulus transfer through tissues in a living organism. Furthermore, the presented study was limited to the use of heparin, while application of more potent thrombolytic drugs could affect the results. We are considering these limitations in our future experiments.

## Conclusion and future work

The presented study evaluated the effectiveness of various types of low frequency mechanical actuation in accelerating reperfusion. A simplified in-vitro model of bloodstream during myocardial infarction was created employing a stenosed, heparinized flow system subject to systemic levels of pressure.

We observed complete reperfusion resulting in TIMI 3 flow in 95% of cases after 11 minutes of vessel deformation at the occlusion site and after 16 minutes of vessel deformation 60 centimeters from the occlusion site, while there was only 2.3% perfusions in the reference system within 20 minutes. The presented results demonstrate that low frequency deformation of blood carrying vessels can be used to accelerate reperfusion, which is of great importance in helping patients suffering from acute myocardial infarction. A simple device can be constructed to apply external mechanical stimulus in the abdomen area of patient’s torso that induces deformation of a major artery, e.g. the aorta. Such method would be non-invasive, safe, inexpensive and suitable for use by inexperienced emergency personnel in the field.

In the future, we plan to continue in-vitro evaluation of the proposed method using various anti-thrombolytic agents dissolved in the buffer fluid and a system that more closely represents the human body. Upon successful verification of this technique, we will proceed with in-vivo testing.

## Competing interests

The authors declare that they have no competing interests.

## Authors’ contributions

MM, BeK and CM conceived the concept and designed the study. Experiments were performed by MM, BeK, FK, KT and MSN. MM and BeK performed the data analysis and interpretation. MM and BeK wrote the manuscript. All authors read and approved the final manuscript.
